# Global, regional, and national burden of oral cancer and its attributable risk factors from 1990 to 2019

**DOI:** 10.1002/cam4.6025

**Published:** 2023-05-02

**Authors:** Rongyin Sun, Weijie Dou, Weiliang Liu, Jin Li, Xiangxiang Han, Shunhang Li, Xueqian Wu, Fei Wang, Xin Xu, Jing Li

**Affiliations:** ^1^ School of Stomatology Weifang Medical University Weifang People's Republic of China; ^2^ School of Health Care Security First Medical University & Shandong Academy of Medical Sciences Jinan People's Republic of China; ^3^ Institute of Health Education and Promotion, Shandong Center for Disease Control and Prevention Jinan People's Republic of China; ^4^ Weifang People's Hospital Weifang People's Republic of China; ^5^ School of Public Health Weifang Medical University Weifang People's Republic of China

**Keywords:** burden of disease, disability‐adjusted life years, incidence, mortality, oral cancer, risk factors

## Abstract

**Background:**

This study aims to provide a theoretical basis for the prevention of oral cancer by analyzing the epidemiological trends of oral cancer.

**Materials and Methods:**

The data on oral cancer from 1990 to 2019 were extracted from the Global Burden of Disease 2019 database. The incidence, mortality, disability‐adjusted life years (DALYs), and age‐standardized rate as well as attributable risk factors of oral cancer were used for the analysis. Estimated annual percentage change (EAPC) was calculated to describe the changes in age‐standardized incidence rate (ASIR), age‐standardized mortality rate (ASMR), and age‐standardized DALYs rate (ASDR).

**Results:**

The global ASIR of oral cancer showed an increasing trend from 1990 to 2019. ASIR in high SDI regions showed a decreasing trend during the studied period, with high SDI regions having the lowest ASMR in 2019. In 2019, the highest ASIR, ASMR, and ASDR were detected in South Asia. At the national level, Pakistan had the highest ASMR and ASDR in 2019. The increasing disease burden was observed in younger populations aged below 45 during the studied period. Smoking and alcohol use still exerted profound impacts on the oral cancer burden, with South Asia having the greatest increase in the percentage of deaths due to oral cancer attributable to chewing tobacco from 1990 to 2019.

**Conclusion:**

In conclusion, there is a large variability in the temporal and spatial burden of oral cancer, and it is essential for priority countries to take targeted intervention policies and measures to reduce the disease burden of oral cancer. In addition, the oral cancer burden caused by attributable risk factors should also receive close attention.

## 1 INTRODUCTION

As one of the most common malignancies occurring in the head and neck, the global epidemiological trends of oral cancer are changing significantly.^1^ The global estimated number of new cases of oral cancer increased from 354,864 reported by GLOBOCAN 2018 to 377,173 reported by GLOBOCAN 2020, and the number of new deaths remained stable at approximately 177,000.^2^, ^3^ While the clinical symptoms and treatments of oral cancer can reduce the quality of life of patients and cause serious economic and psychological burdens.^4^

Noticeable geographic heterogeneity exists in the disease burden of oral cancer. A high prevalence of oral cancer has been observed in South and Southeast Asia.^5^ It has been reported that India accounted for about one‐third of the total global burden of oral cancer.^6^ The economic level also has a substantial impact on the disease burden of oral cancer. Compared with developed countries, the burden in developing countries are more serious.^7^ Low socioeconomic status can add to the incidence risk of oral cancer, similar to the impact of lifestyle risk factors.^8^ However, the prognosis of oral cancer in developed countries is also unsatisfactory, with a five‐year survival rate in the United States being only 65%.^9^

Oral cancer is harmful to both society and individuals, thus it is necessary to conduct a comprehensive and systematic study on the burden of this disease. Although there have been previous studies on the disease burden of oral cancer,^10^, ^11^ few analyses have been performed on the changing trends of attributable risk factors of oral cancer. Investigation of the above trends can help lay a foundation for the formulation of the primary prevention policies for oral cancer.^12^ Thus, the purpose of this study is to analyze the temporal and spatial burden of oral cancer and further evaluate the impact of attributable risk factors on oral cancer, which may facilitate the adoption of targeted measures by local governments to prevent oral cancer.

## 2 MATERIALS AND METHODS

### 2.1 Study data

The data included in this study were extracted from the Global Burden of Disease (GBD) 2019 database, which quantified the health loss caused by 369 diseases, injuries and 87 risk factors in 204 countries and territories.^13^, ^14^ All data were obtained through an online query tool at the GBD website (http://ghdx.healthdata.org/gbd‐results‐tool). Geographically, 21 GBD regions were divided in the GBD study. The socio‐demographic index (SDI) is a comprehensive index composed of income per capita, average educational attainment and fertility rate.^15^ The SDI value ranges from 0 to 1, with lower values indicating lower theoretical level of development related to health outcomes. The countries are divided into five levels (low, low‐middle, middle, high‐middle, and high SDI) based on the SDI values.

International Classification of Diseases (ICD) is used in the GBD study to classify the studied diseases, and the ICD‐10 code for oral cancer included in this study was C00–C08. Oropharyngeal and nasopharyngeal cancers were excluded. Observed indicators included the incidence, mortality and disability‐adjusted life years (DALYs) at the global, regional and national levels from 1990 to 2019, as well as their related age‐standardized rate (ASR). The 95% uncertainty interval (95% UI) was the estimate due to measurement errors and biases.^16^ Annual data on attributable risk factors for deaths and DALYs of oral cancer were also extracted, including smoking, alcohol use, and use of chewing tobacco. The age groups selected were 0–14, 15–19 … 80–84, 85 years and older.

### 2.2 Statistical analysis

The formula for the calculation of the ASR (per 100,000 people) was presented as ASR = $\frac{\sum_{i=1}^{A}\text{aiwi}}{\sum_{i=1}^{A}\mathrm{wi}}\times 100,000$, which could indicate the differences between groups with varying age compositions.^17^ The estimated annual percentage change (EAPC) was calculated to reflect and measure the changing trends of ASR within a specific time period.^18^ Under the linear model, a regression line is consistent with the natural logarithm of ASR, that is, *y* = *α* + *βx* + *ϵ*, with y representing ln(ASR), *x* referring to calendar year, and *ϵ* being the error term. EAPC was calculated as 100 × [exp (*β*)–1], and its 95% confidence interval (95% CI) could be obtained from the linear regression model.^19^ If both EAPC and the lower limit of its 95% CI were greater than 0, the trend of ASR variation would be regarded as upward; on the contrary, if both EAPC and its upper limit 95% CI were lower than 0, the trend of ASR variation would be considered downward. All statistical analyses and data visualization were conducted using the R software (version 4.2.1).

## 3 RESULTS

### 3.1 Incidence of oral cancer

At the global level, the age‐standardized incidence rate (ASIR) of oral cancer increased slightly from 4.28/100,000 to 4.52/100,000 from 1990 to 2019. Compared with that in 1990, the global ASIR of males decreased and of females increased in 2019. The incident cases of oral cancer in the world and all GBD regions has increased in different proportions during the period (Table 1).

**TABLE 1:** The incident cases and ASIR of oral cancer in 1990 and 2019.

Location	1990		2019	
	Incident cases (×10^3^)	ASIR (1/10^5^)	Incident cases (×10^3^)	ASIR (1/10^5^)
Global	175.63 (167.52–184.91)	4.28 (4.07–4.51)	373.10 (340.88–403.87)	4.52 (4.13–4.89)
Sex				
Male	120.66 (112.73–129.49)	6.24 (5.84–6.70)	243.19 (218.65–268.26)	6.16 (5.55–6.19)
Female	54.97 (51.32–58.50)	2.54 (2.37–2.70)	129.91 (117.07–142.96)	3.01 (2.71–3.31)
SDI level				
Low SDI	12.33 (10.40–14.36)	4.75 (3.99–5.54)	30.84 (27.23–34.74)	5.36 (4.76–6.00)
Low‐middle SDI	38.07 (33.58–43.11)	5.88 (5.15–6.68)	95.60 (83.27–10.83)	6.65 (5.81–7.52)
Middle SDI	31.47 (29.34–33.56)	2.87 (2.68–3.06)	94.42 (82.94–105.84)	3.72 (3.28–4.16)
High‐middle SDI	38.60 (37.41–39.79)	3.55 (3.44–3.66)	71.61 (64.77–78.12)	3.56 (3.22–3.88)
High SDI	55.08 (53.52–56.28)	5.50 (5.35–5.61)	80.46 (72.63–88.74)	4.71 (4.26–5.20)
Region				
Andean Latin America	0.31 (0.27–0.35)	1.43 (1.26–1.61)	0.87 (0.70–1.05)	1.53 (1.24–1.86)
Australasia	1.53 (1.46–1.60)	6.67 (6.36–6.98)	1.84 (1.50–2.25)	4.05 (3.27–4.95)
Caribbean	1.09 (1.02–1.16)	4.14 (3.88–4.43)	2.10 (1.79–2.45)	4.05 (3.45–4.73)
Central Asia	1.35 (1.26–1.52)	2.74 (2.54–3.11)	2.41 (2.18–2.67)	3.06 (2.78–3.40)
Central Europe	6.96 (6.77–7.14)	4.79 (4.66–4.92)	10.34 (8.98–11.72)	5.35 (4.66–6.07)
Central Latin America	1.66 (1.60–1.70)	1.94 (1.86–2.00)	4.25 (3.63–4.93)	1.80 (1.54–2.09)
Central Sub‐Saharan Africa	0.64 (0.49–0.81)	2.63 (1.97–3.30)	1.52 (1.15–1.93)	2.64 (1.98–3.34)
East Asia	13.86 (12.34–15.53)	1.50 (1.34–1.67)	52.04 (44.27–61.05)	2.50 (2.13–2.92)
Eastern Europe	9.20 (8.68–9.79)	3.29 (3.11–3.51)	12.96 (11.61–14.51)	3.98 (3.56–4.46)
Eastern Sub‐Saharan Africa	2.47 (2.09–2.92)	2.92 (2.46–3.45)	6.12 (5.13–7.09)	3.28 (2.77–3.74)
High‐income Asia Pacific	3.57 (3.44–3.67)	1.78 (1.71–1.84)	8.48 (7.21–9.69)	2.07 (1.79–2.36)
High‐income North America	2.34 (2.27–2.40)	6.98 (6.78–7.14)	33.20 (28.79–38.39)	5.60 (4.85–6.49)
North Africa and Middle East	2.42 (2.03–2.79)	1.33 (1.12–1.54)	6.65 (5.81–7.69)	1.46 (1.28–1.68)
Oceania	0.10 (0.08–0.13)	2.87 (2.25–3.71)	0.25 (0.19–0.34)	3.17 (2.49–4.15)
South Asia	54.22 (47.97–61.09)	8.82 (7.71–10.01)	143.20 (120.85–166.17)	9.65 (8.17–11.15)
Southeast Asia	11.13 (10.02–12.07)	4.13 (3.70–4.49)	28.95 (24.16–34.65)	4.70 (3.94–5.65)
Southern Latin America	1.29 (1.23–1.35)	2.78 (2.66–2.91)	2.14 (1.68–2.68)	2.62 (2.06–3.30)
Southern Sub‐Saharan Africa	1.18 (1.05–1.39)	4.01 (3.54–4.78)	2.21 (2.01–2.44)	3.72 (3.39–4.10)
Tropical Latin America	3.95 (3.81–4.08)	4.11 (3.94–4.25)	9.74 (9.16–10.24)	3.94 (3.71–4.15)
Western Europe	34.03 (32.99–34.96)	6.48 (6.29–6.66)	40.62 (35.16–46.78)	5.17 (4.46–5.96)
Western Sub‐Saharan Africa	1.24 (1.05–1.42)	1.34 (1.15–1.53)	3.21 (2.71–3.75)	1.60 (1.38–1.84)

*Note*: Incident cases (95% uncertainty interval).

Abbreviations: ASIR: age‐standardized incidence rate (95% uncertainty interval).

**FIGURE 1:**
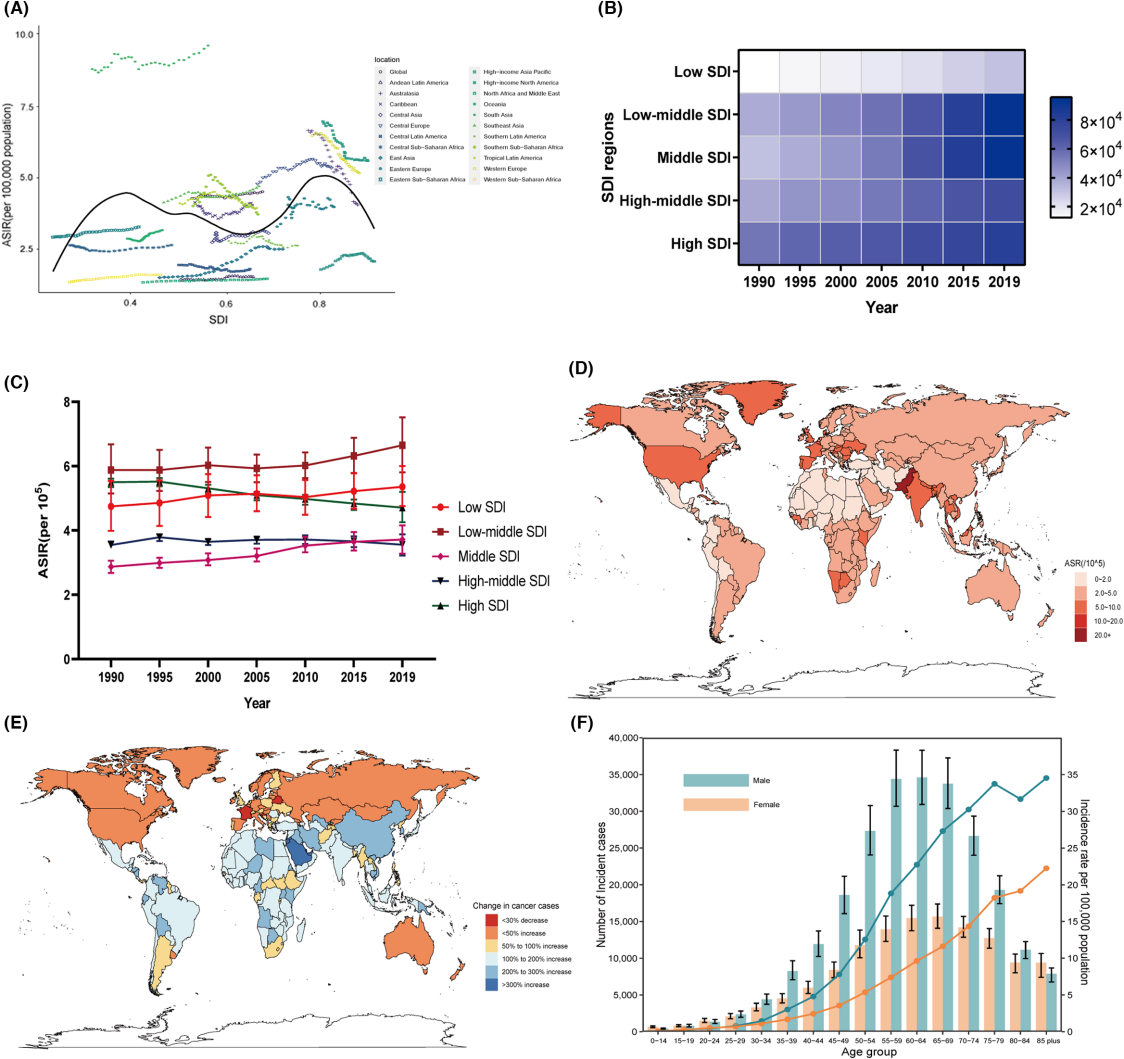
The incidence of oral cancer. (A) the trend of ASIR by SDI from 1990 to 2019 at the global and regional levels; (B,C) the trends of incident cases and ASIR in five SDI regions from 1990 to 2019; (D) the ASIR in 204 countries and territories in 2019; (E) The change in incident cases of oral cancer over the past 30 years; (F) global incident cases and ASIR of oral cancer by age and sex in 2019. ASIR, age‐standardized incidence rate. SDI, socio‐demographic index.

The highest ASIR was observed in South Asia (9.65/100,000) in 2019, which was approximately twice as that in North America, where the economic status is higher (Figure 1A). The incident cases of oral cancer in five SDI regions showed an increasing trend from 1990 to 2019 (Figure 1B), and only the ASIR in high SDI regions showed a decreasing trend during the studied period. Low SDI regions (6.65/100,000) had the highest ASIR of oral cancer in 2019 (Figure 1C). At the national level, Palau (29.85/100,000) had the highest ASIR in 2019, followed by Pakistan (Figure 1D). Changes in incident cases in 204 countries and territories between 1990 and 2019 are presented in Figure 1E. Global incident cases and ASIR in 2019 are exhibited by sex and age groups in Figure 1F. The highest incident cases of oral cancer in males and females were in the 60‐ to 64‐year‐old and 65‐ to 69‐year‐old groups, respectively. Compared with those in 1990, the global incident cases and ASIR in individuals aged below 45 increased in 2019 (Table S3).

### 3.2 Mortality of oral cancer

Globally, the deaths caused by oral cancer in 2019 (199.40 × 10^3^) were more than twice as many as those in 1990 (96.63 × 10^3^). Deaths due to oral cancer in all GBD regions also showed increasing trends during the studied period. However, the global age‐standardized mortality rate (ASMR) in 1990 and 2019 remained stable. Compared with that in 1990, the global ASMR of males and females in 2019 decreased and increased, respectively (Table S1).

**FIGURE 2:**
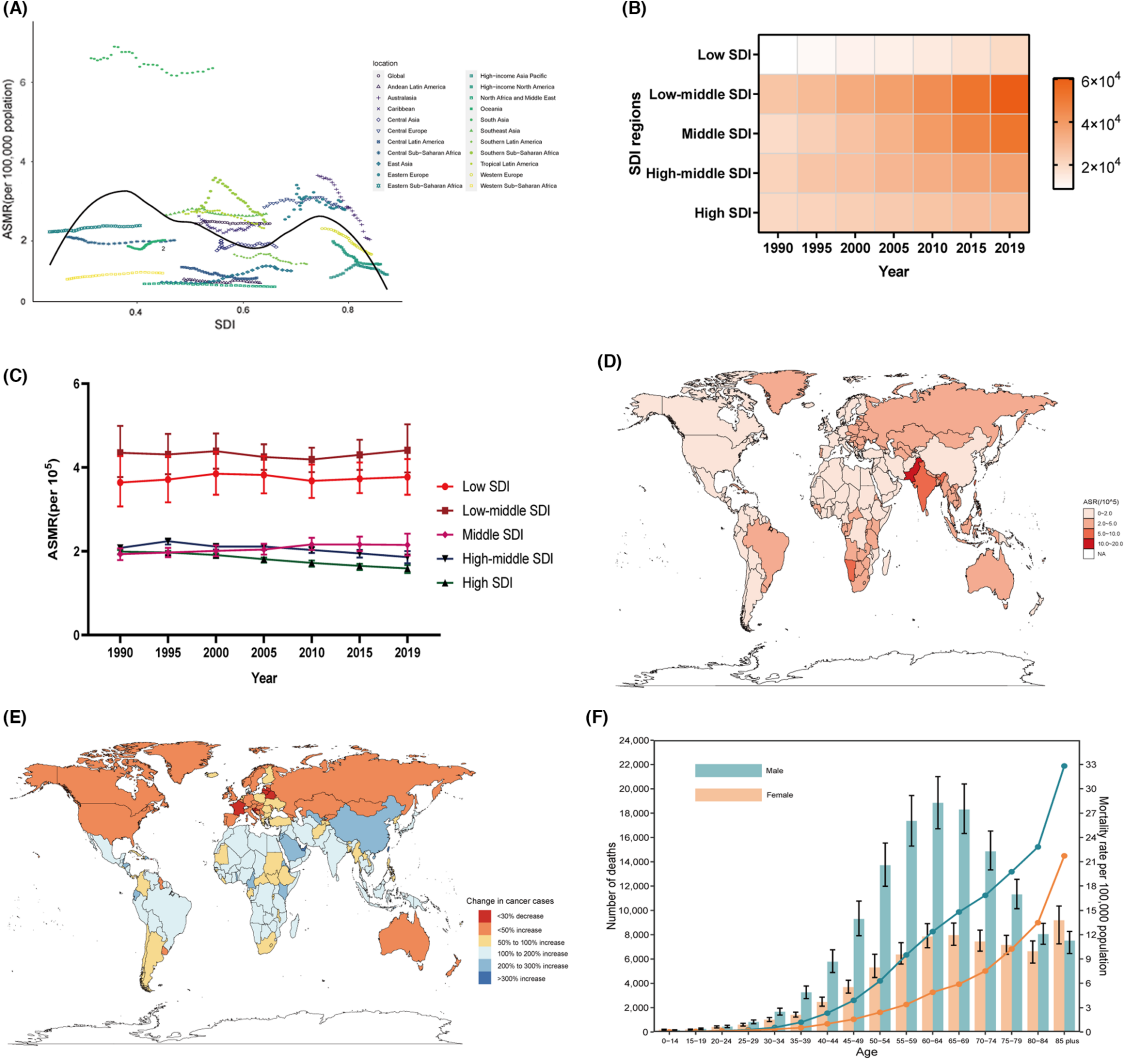
The mortality of oral cancer. (A) the trend of ASMR by SDI from 1990 to 2019 at the global and regional levels; (B,C) the trends of deaths and ASMR in five SDI regions from 1990 to 2019; (D) the ASMR in 204 countries and territories in 2019; (E) The change in deaths of oral cancer over the past 30 years; (F) global deaths and ASMR of oral cancer by age and sex in 2019. ASMR, age‐standardized mortality rate. SDI, socio‐demographic index.

South Asia (6.36/100,000) had the highest ASMR of oral cancer in 2019, followed by Central Europe (Figure 2A). Deaths due to oral cancer in five SDI regions showed increasing trends from 1990 to 2019 (Figure 2B), and the lowest ASMR of oral cancer was found in high SDI regions (1.59/100,000) in 2019 (Figure 2C). At the national level, ASMR in almost a quarter of the countries and territories exceeded the global average level in 2019, with Pakistan (14.72/100,000) having the highest ASMR, followed by Palau (Figure 2D). Variations in the number of deaths due to oral cancer in the 204 countries and territories from 1990 to 2019 are presented in Figure 2E. In 2019, more deaths and a higher ASMR were found in males than females in most age groups (Figure 2F). Compared with those in 1990, the global deaths and ASMR in individuals aged below 45 remained stable or increased in 2019 (Table S4).

### 3.3 DALYs of oral cancer

At the global level, DALYs of oral cancer increased by 92.89% (95% UI: 67.78%–117.64%) from 1990 to 2019, and increasing trends of the DALYs were observed in all GBD regions except Western Europe. The age‐standardized DALYs rate (ASDR) of oral cancer decreased from 67.01/100,000 in 1990 to 66.05/100,000 in 2019. Compared with that in 1990, the global ASDR of males decreased and of females increased in 2019 (Table S2).

**FIGURE 3:**
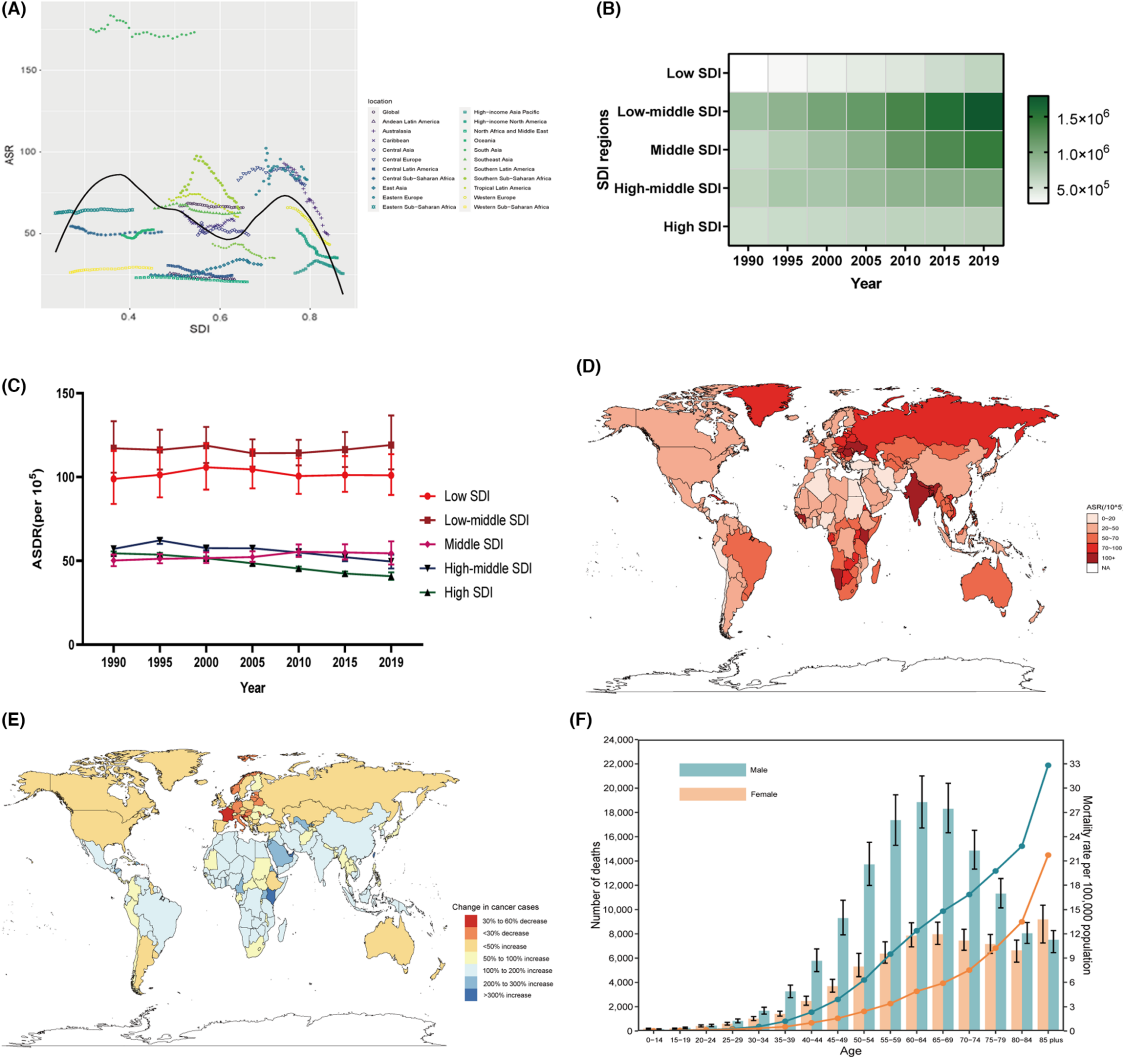
The DALYs of oral cancer. (A) the trend of ASDR by SDI from 1990 to 2019 at the global and regional levels; (B,C) the trends of DALYs and ASDR in five SDI regions from 1990 to 2019; (D) the ASDR in 204 countries and territories in 2019; (E) The change in DALYs of oral cancer over the past 30 years; (F) global DALYs and ASDR of oral cancer by age and sex in 2019. ASDR, age‐standardized DALYs rate. SDI: socio‐demographic index.

The highest ASDR was observed in South Asia (173.17/100,000) in 2019, nearly five times that in North America, where the economic status is higher (Figure 3A). The DALYs of oral cancer in five SDI regions showed increasing trends from 1990 to 2019 (Figure 3B). The ASDR in high‐middle SDI and high SDI regions decreased during the studied period, and high SDI regions (40.83/100,000) had the lowest ASDR in 2019 (Figure 3C). At the national level, Pakistan (421.87/100,000) had the highest ASDR in 2019, followed by Palau (Figure 3D). Variations in oral cancer DALYs in the 204 countries and territories during the studied period are displayed in Figure 3E. The ASDR in males peaked at the age of 65–69 years, while the ASDR in females increased with age (Figure 3F). Compared with those in 1990, the global DALYs and ASDR in individuals aged below 45 also increased in 2019 (Table S5).

### 3.4 EAPC in incidence, mortality, and DALYs

From 1990 to 2019, the global EAPC of oral cancer was 0.14% in ASIR (Figure S1A), −0.05% in ASMR (Figure S2A), and − 0.12% in ASDR (Figure S3A). The highest EAPCs in ASIR, ASMR and ASDR were found in East Asia, indicating that this area had the greatest increase in ASR during the studied period. Significant differences were observed in the EAPC of ASIR, ASMR and ASDR in the 204 countries and territories (Figures S1B–S3B), with Cabo Verde having the highest EAPC of ASIR, ASMR, and ASDR, at 5.11%, 4.64%, and 4.75%, respectively; the lowest EAPC was observed in Mongolia.

### 3.5 Attributable risk factors

At the global level, the percentage of oral cancer deaths attributable to both smoking and alcohol use showed decreasing trends from 1990 to 2019, and the percentage of oral cancer deaths attributable to the use of chewing tobacco increased from 15.95% in 1990 to 18.70% in 2019 (Figure S4A,B). Compared with that in 1990, except for East Asia and Eastern Europe, the percentage of oral cancer deaths attributable to smoking in all GBD regions and SDI regions showed downward trends in 2019. Through the studied period, the percentage of oral cancer deaths attributable to alcohol use decreased in high‐middle and high SDI regions. The greatest increase in the percentage of oral cancer deaths attributable to the use of chewing tobacco was found in South Asia from 1990 to 2019.

The global DALYs of oral cancer was also affected by the above risk factors in 2019, with 30.08% attributable to smoking, 31.36% attributable to alcohol use and 18.66% attributable to the use of chewing tobacco. In addition, during the studied period, East Asia and Southeast Asia have witnessed the greatest increase in the percentage of oral cancer DALYs attributable to smoking and alcohol use, respectively. In 2019, the highest percentage of oral cancer DALYs attributable to the use of chewing tobacco was observed in South Asia.

## 4 DISCUSSION

The present study revealed that the disease burden of oral cancer has changed substantially from 1990 to 2019. From a global perspective, the incident cases and ASIR of oral cancer showed an upward trend during the studied period. The development of screening technologies such as salivary biomarkers has contributed to the early detection of oral cancer,^20^, ^21^ so that the accurate identification of patients who have not been diagnosed with oral cancer before may explain the above results. On the other hand, the global ASMR and ASDR of oral cancer were stable or showed decreasing trends, which might be related to the development of multidisciplinary treatment strategies and the application of adjuvant therapies, which can significantly improve the prognosis of oral cancer.^22^, ^23^

At the regional and national levels, serious disease burden of oral cancer was observed in Asia. South Asia had the largest number of incident cases, deaths and DALYs as well as the highest corresponding ASR in 2019, and the increasing burden of oral cancer in India played a key role in this during the studied period. A study using statistics from the international agency for research on cancer (IARC) has also demonstrated that India and Pakistan had a high burden of oral cancer.^24^

It can be found that tobacco (including smoking and smokeless) and alcohol use are significant attributable risk factors for oral cancer in Asia,^4^ with South Asian countries maintaining a high tobacco consumption rate. In addition to smoking, smokeless tobacco (SLT) also has oral carcinogenicity.^25^ The main burden of SLT is concentrated in South Asian countries, with India being one of the countries bearing the dual burden of smoking and SLT.^26^ Chewing tobacco is a form of SLT. The present study indicated that the use of chewing tobacco substantially contributed to the deaths and DALYs due to oral cancer in South Asia. A previous study reported that 83.29% of global chewing tobacco users were observed in South Asia in 2019, and the prevalence rate has remained high.^27^ It has also been reported that smoking and alcohol consumption were significantly associated with higher susceptibility to oral cancer in people in East Asia,^28^ with smoking and alcohol consumption acting independently and synergistically on increasing the incidence risk of oral cancer.^29^ Involuntary smoking may also be strongly associated with oral cancer in East Asian individuals.^30^

In addition to the above attributable risk factors, areca nut is another well‐known risk factor leading to oral cancer. As the fourth most common addictive substance in the world,^31^ areca nut has been listed as Class I carcinogen by IARC. The habit of chewing areca nuts, which can cause oral precancerous lesions that may deteriorate into oral cancer, is prevalent in many Asia Pacific countries.^32^, ^33^ The risk of oral cancer increases with the increase of time and frequency of chewing areca nuts, regardless of the use of tobacco on top of it.^34^ Since many people are becoming dependent on areca nuts and there has been no policy to restrict the use of areca nuts, it is critical for all countries to develop strategies regarding this addictive and harmful substance.^35^ Moreover, a meta‐analysis suggested that there was a significant correlation between human papilloma virus and oral cancer.^36^ Therefore, to avoid the harm caused by attributable risk factors, it is necessary for individuals to develop good living habits and improve their awareness of oral cancer prevention.

As population growth and aging has become a more serious concern and specific risk factors has become more prevalent, the burden of cancer has become more alarming.^37^ It is anticipated that, from 2020 to 2070, the number of patients with cancer worldwide will continue to rise, with the greatest increase being in low‐income countries.^38^ As shown in the present study, the disease burden of oral cancer was negatively correlated with the level of SDI, indicating that in countries with low socioeconomic status, the disease burden of oral cancer is unproportional to the economic level.^39^

Furthermore, the most serious burden of oral cancer caused by tobacco has been found in low‐and middle‐income countries.^40^ This is closely related to the poor awareness of the attributable risk factors for oral cancer among people in these countries.^41^ On the other hand, many low‐and middle‐income countries have difficulties in implementing the WHO Framework Convention on Tobacco Control, leading to failure to achieve goals of scheduled tobacco control.^42^ Thus, the increasing prevalence of smoking will undoubtedly increase the burden of oral cancer in these countries.

The present study also showed that the burden of oral cancer was gradually increasing in the younger population. A retrospective study revealed that the incidence of oral cancer in the younger population was on the rise, indicating that the etiology and clinical features of young patients might be unique.^43^ Moreover, the burden of oral cancer among females showed an increasing trend in the present study, which might be partly related to the habits of tobacco and alcohol use among females.^44^

Despite the strengths of the present study, limitations are inevitable. First, the data of lip cancer and oral cancer are not classified separately in the GBD database, which might have led to the overestimation of the incidence of oral cancer. Second, as the number of attributable risk factors of oral cancer is limited in the GBD database, the impact of other risk factors on oral cancer could not be quantitatively analyzed.

In conclusion, oral cancer is still a prominent global public health problem due to the increasing disease burden. The burden of oral cancer in females and the younger population aged below 45 years has been on the rise; thus, attention should also be paid to them while concerning about the key population for oral cancer control. As there is a significant negative correlation between the burden of oral cancer and the SDI level, interventions against attributable risk factors need to be implemented based on the actual situations in different countries and regions. It is essential to improve the public awareness of primary oral cancer prevention and develop the economy, in order to control attributable risk factors and eventually reduce the oral cancer burden.

## AUTHOR CONTRIBUTIONS

**Rongyin Sun:** Conceptualization (lead); methodology (lead); writing – original draft (lead); writing – review and editing (equal). **Weijie Dou:** Conceptualization (lead); methodology (lead); Writing – review and editing (equal). **Weiliang Liu:** Conceptualization (equal); methodology (equal); Writing – review and editing (equal). **Jin Li:** methodology (equal); writing – original draft (equal). **Xiangxiang Han:** methodology (equal); writing – original draft (equal). **Shunhang Li:** Formal analysis (lead); software (lead). **Xueqian Wu:** Data curation (equal). **Fei Wang:** Data curation (equal). **Xin Xu:** Conceptualization (lead); supervision (equal); writing – review and editing (lead). **Jing Li:** Conceptualization (lead); supervision (equal); writing – review and editing (lead).

## Supporting information


Figure S1.

Figure S2.

Figure S3.

Figure S4.

**Table S1.** **Table S2**. **Table S3**. **Table S4**. **Table S5**.

## Data Availability

The datasets analyzed for this study can be found in the Global Burden of Disease database [http://ghdx.healthdata.org/gbd‐results‐tool].
