# Supplementary figures and images for: Global, regional, and national burden of oral cancer and its attributable risk factors from 1990 to 2019
Source: Cancer Med. 2023 May 2;12(12):13811–20. doi: 10.1002/cam4.6025 (PMC10315711; doi:10.1002/cam4.6025)

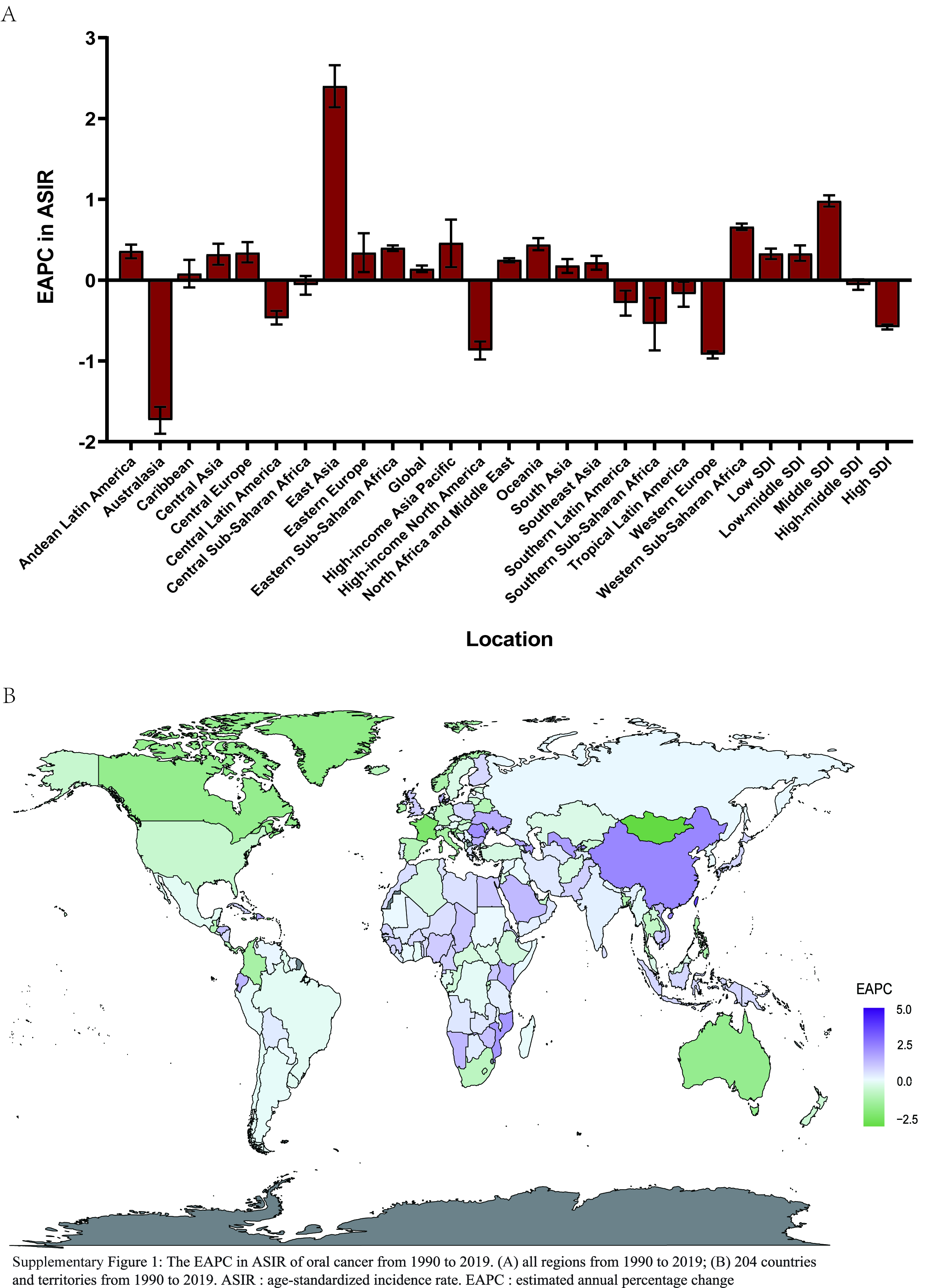

Supplement: Supplementary file 1 — Figure S1. [file CAM4-12-13811-s001.tif]

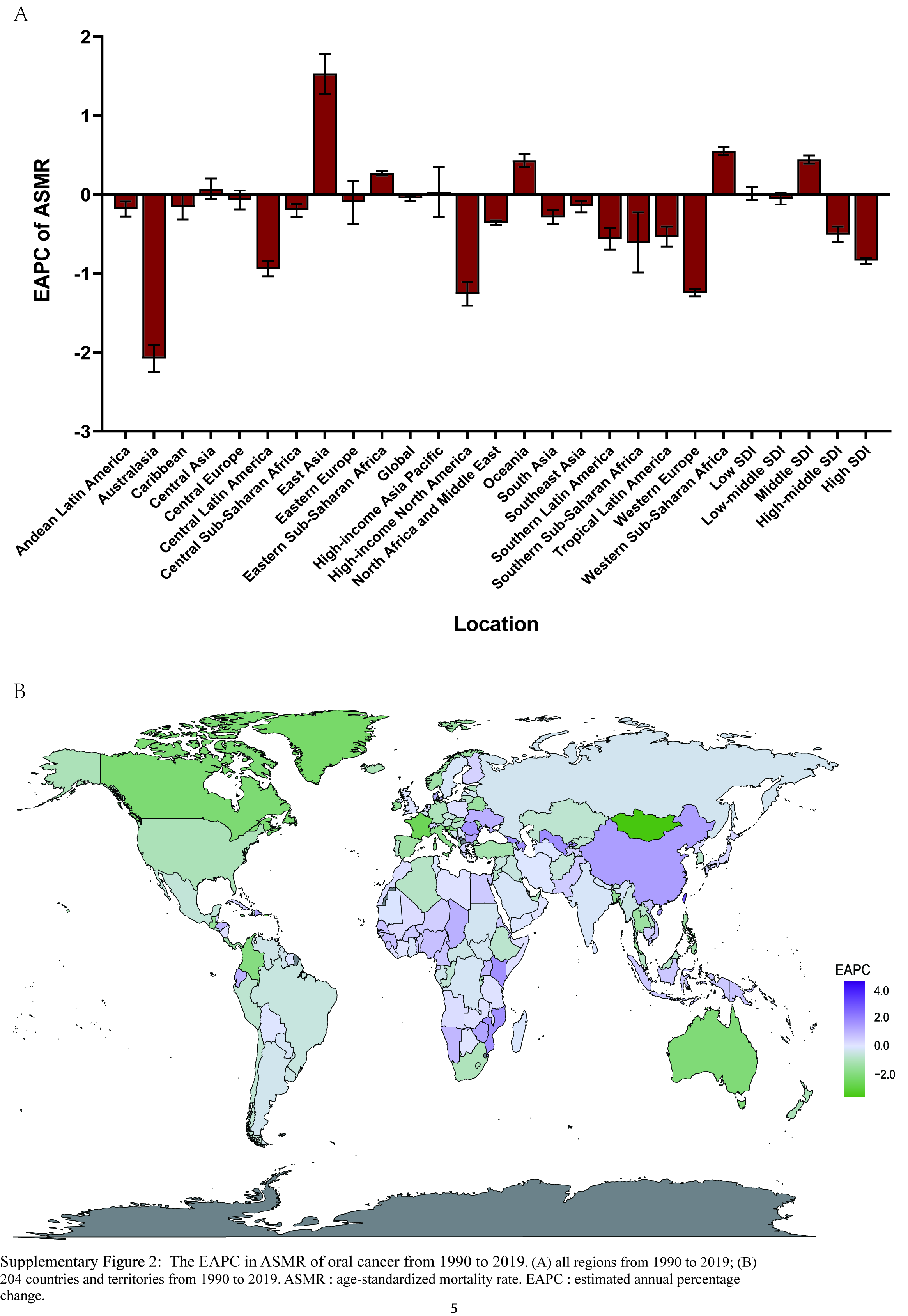

Supplement: Supplementary file 2 — Figure S2. [file CAM4-12-13811-s002.tif]

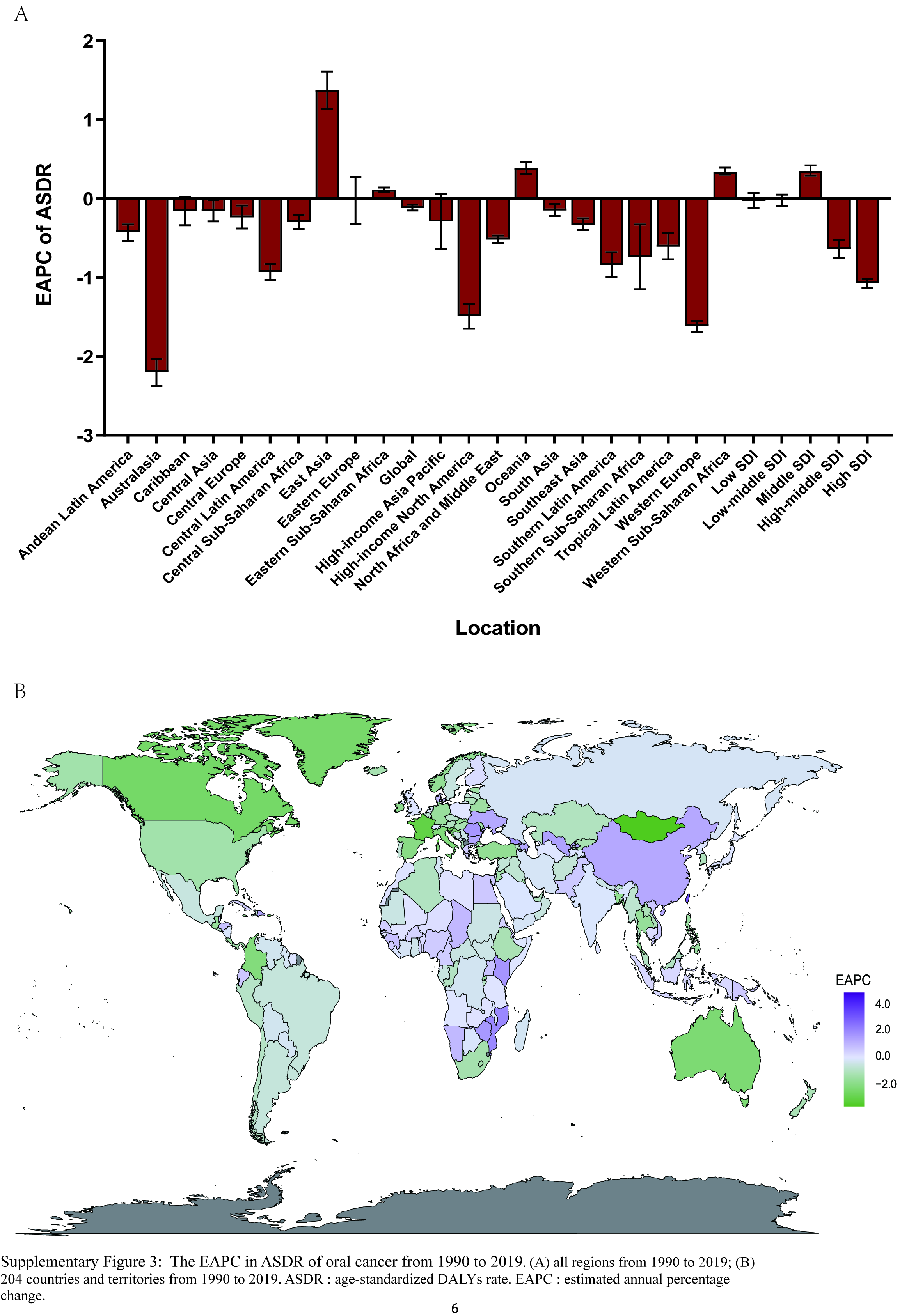

Supplement: Supplementary file 3 — Figure S3. [file CAM4-12-13811-s004.tif]

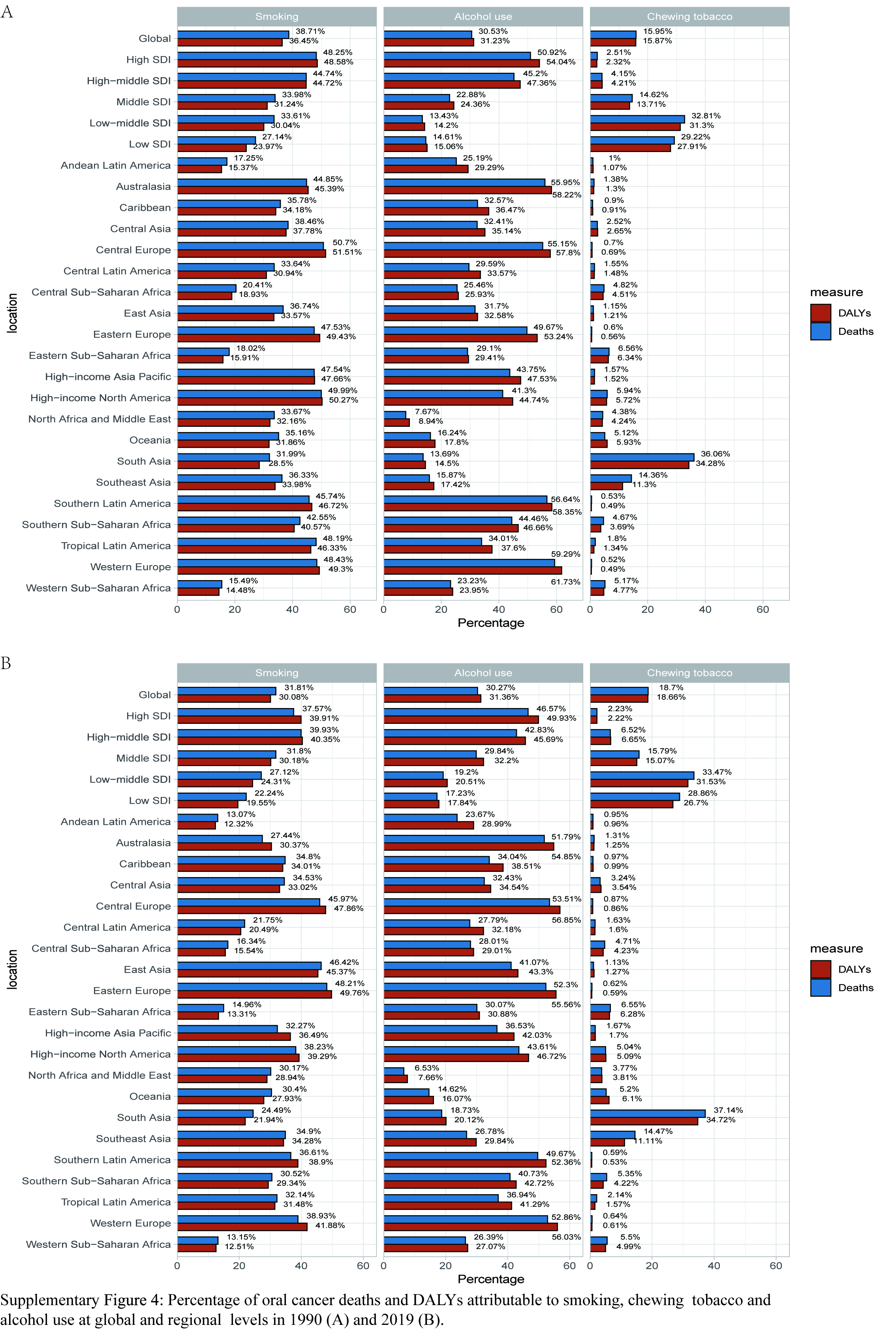

Supplement: Supplementary file 4 — Figure S4. [file CAM4-12-13811-s003.tif]
