# Supplementary material for: Global, regional, and national burden of oral cancer and its attributable risk factors from 1990 to 2019
Source: Cancer Med. 2023 May 2;12(12):13811–20. doi: 10.1002/cam4.6025 (PMC10315711; doi:10.1002/cam4.6025)
Supplement: Supplementary file 5 — Table S1. Table S2. Table S3. Table S4. Table S5. [file CAM4-12-13811-s005.docx]

| Supplementary Table 1. The Deaths and ASMR of oral cancer in 1990 and 2019. | | | | | |
| --- | --- | --- | --- | --- | --- |
| Location | 1990 | |  | 2019 | |
|  | Deaths (×103) | ASMR (1/105) |  | Deaths (×103) | ASMR (1/105) |
| Global | 96.63 (90.59-103.05) | 2.44 (2.28-2.60) |  | 199.40 (181.65-218.06) | 2.44 (2.22-2.66) |
| Sex |  |  |  |  |  |
| Male | 66.99 (61.24-73.05) | 3.63 (3.32-3.95) |  | 131.56 (117.70-145.46) | 3.42 (3.06-3.77) |
| Female | 29.63 (27.30-31.84) | 1.41 (1.30-1.52) |  | 67.84 (60.78-75.65) | 1.56 (1.40-1.74) |
| SDI category |  |  |  |  |  |
| Low SDI | 8.76 (7.41-10.26) | 3.64 (3.07-4.27) |  | 30.84 (27.23-34.74) | 3.77 (3.35-4.20) |
| Low-middle SDI | 26.23 (22.97-29.89) | 4.35 (3.77-4.99) |  | 60.33 (53.05-68.96) | 4.41 (3.88-5.03) |
| Middle SDI | 19.42 (18.11-20.77) | 1.93 (1.79-2.07) |  | 52.14 (46.08-58.78) | 2.15 (1.90-2.42) |
| High-middle SDI | 21.99 (21.24-22.70) | 2.08 (2.00-2.15) |  | 37.50 (34.56-40.49) | 1.86 (1.71-2.00) |
| High SDI | 20.18 (19.53-20.56) | 1.99 (1.93-2.03) |  | 29.36 (27.06-30.97) | 1.59 (1.48-1.67) |
| Region |  |  |  |  |  |
| Andean Latin America | 0.21 (0.19-0.24) | 1.04 (0.91-1.16) |  | 0.52 (0.42-0.63) | 0.94 (0.77-1.14) |
| Australasia | 0.84 (0.80-0.87) | 3.65 (3.49-3.78) |  | 1.00 (0.90-1.08) | 2.04 (1.85-2.19) |
| Caribbean | 0.67 (0.62-0.72) | 2.63 (2.44-2.84) |  | 1.24 (1.06-1.44) | 2.39 (2.05-2.77) |
| Central Asia | 0.84 (0.78-0.96) | 1.76 (1.63-2.02) |  | 1.38 (1.25-1.53) | 1.85 (1.68-2.04) |
| Central Europe | 6.96 (6.77-7.14) | 4.79 (4.66-4.92) |  | 10.34 (8.98-11.72) | 5.35 (4.66-6.07) |
| Central Latin America | 1.06 (1.02-1.09) | 1.34 (1.27-1.38) |  | 2.48 (2.14-2.88) | 1.07 (0.92-1.24) |
| Central Sub-Saharan Africa | 0.47 (0.36-0.59) | 2.10 (1.57-2.61) |  | 1.06 (0.80-1.35) | 2.02 (1.50-2.55) |
| East Asia | 8.02 (7.04-9.00) | 0.96 (0.85-1.07) |  | 25.27 (21.55-29.68) | 1.25 (1.06-1.45) |
| Eastern Europe | 7.08 (6.72-7.49) | 2.54 (2.42-2.69) |  | 9.22 (8.16-10.37) | 2.79 (2.47-3.13) |
| Eastern Sub-Saharan Africa | 1.76 (1.47-2.09) | 2.23 (1.87-2.63) |  | 4.08 (3.46-4.70) | 2.39 (2.04-2.71) |
| High-income Asia Pacific | 2.09 (2.00-2.14) | 1.08 (1.02-1.11) |  | 5.55 (4.71-6.03) | 1.15 (1.02-1.23) |
| High-income North America | 6.83 (6.57-7.00) | 1.99 (1.92-2.04) |  | 8.90 (8.38-9.25) | 1.42 (1.34-1.47) |
| North Africa and Middle East | 1.53 (1.28-1.77) | 0.92 (0.77-1.07) |  | 3.54 (3.10-4.09) | 0.85 (0.75-0.97) |
| Oceania | 0.05 (0.04-0.07) | 1.87 (1.45-2.44) |  | 0.14 (0.11-0.19) | 2.02 (1.59-2.66) |
| South Asia | 37.27 (32.32-42.35) | 6.61 (5.73-7.55) |  | 89.34 (76.99-104.30) | 6.36 (5.48-7.39) |
| Southeast Asia | 6.46 (5.80-6.98) | 2.65 (2.36-2.86) |  | 15.33 (12.91-18.27) | 2.68 (2.26-3.19) |
| Southern Latin America | 0.76 (0.73-0.79) | 1.66 (1.59-1.72) |  | 1.17 (1.09-1.25) | 1.42 (1.32-1.51) |
| Southern Sub-Saharan Africa | 0.77 (0.67-0.91) | 2.76 (2.40-3.31) |  | 1.40 (1.28-1.54) | 2.48 (2.29-2.73) |
| Tropical Latin America | 2.46 (2.37-2.55) | 2.72 (2.59-2.83) |  | 5.65 (5.29-5.97) | 2.32 (2.17-2.46) |
| Western Europe | 12.49 (12.08-12.77) | 2.31 (2.24-2.36) |  | 14.40 (13.34-15.07) | 1.66 (1.57-1.73) |
| Western Sub-Saharan Africa | 0.89 (0.77-1.03) | 1.04 (0.89-1.19) |  | 2.17 (1.85-2.52) | 1.19 (1.03-1.36) |
| Deaths (95% uncertainty interval), ASMR: age-standardized mortality rate (95% uncertainty interval) | | | | | |

| Supplementary Table 2. The DALYs and ASDR of oral cancer in 1990 and 2019. | | | | | |
| --- | --- | --- | --- | --- | --- |
| Location | 1990 | |  | 2019 | |
|  | DALYs (×10^3^) | ASDR (1/10^5^) |  | DALYs (×10^3^) | ASDR (1/10^5^) |
| Global | 2854.78 (2680.93-3052.61) | 67.01 (62.93-71.57) |  | 5506.65 (5004.33-6033.42) | 66.05 (60.06-72.35) |
| Sex |  |  |  |  |  |
| Male | 2027.83 (1850.00-2216.49) | 98.85 (90.26-107.85) |  | 3759.37 (3330.03-4181.24) | 92.84 (82.41-103.07) |
| Female | 826.95 (759.91-893.27) | 37.41 (34.45-40.33) |  | 1747.29 (1564.23-1951.57) | 40.78 (36.48-45.55) |
| SDI category |  |  |  |  |  |
| Low SDI | 278.27 (236.87-324.37) | 98.91 (83.87-115.74) |  | 628.76 (553.38-714.29) | 101.02(89.36-113.63) |
| Low-middle SDI | 817.72 (718.90-927.96) | 117.11 (102.64-133.3) |  | 1787.73 (1570.40-2051.59) | 119.12 (104.60-136.74) |
| Middle SDI | 589.19 (549.38-630.76) | 50.25 (46.81-53.81) |  | 1428.52 (1253.14-1618.15) | 54.45 (47.87-61.67) |
| High-middle SDI | 634.20 (612.34-656.61) | 57.15 (55.15-59.16) |  | 991.69 (909.23-1072.35) | 49.58 (45.43-53.65) |
| High SDI | 534.13 (521.67-545.83) | 54.49 (53.26-55.65) |  | 667.69 (630.37-703.75) | 40.83 (38.68-43.14) |
| Region |  |  |  |  |  |
| Andean Latin America | 5.94 (5.23-6.69) | 25.84 (22.7-29.02) |  | 12.73(10.21-15.66) | 22.04(17.74-26.97) |
| Australasia | 21.13 (20.39-21.86) | 93.07 (89.8-96.28) |  | 21.71(20.03-23.30) | 49.42(45.94-52.98) |
| Caribbean | 17.01 (15.74-18.47) | 63.45 (58.82-69.02) |  | 30.09(25.69-35.23) | 58.00(49.52-67.85) |
| Central Asia | 25.24 (23.50-28.34) | 49.25 (45.88-55.54) |  | 41.63(37.57-46.48) | 49.31(44.63-54.77) |
| Central Europe | 119.65 (116.74-122.48) | 82.87 (80.88-84.76) |  | 146.44(127.31-165.80) | 79.77(69.17-90.48) |
| Central Latin America | 27.99 (27.18-28.64) | 30.65 (29.63-31.46) |  | 59.37(51.02-69.45) | 24.62(21.21-28.79) |
| Central Sub-Saharan Africa | 14.68 (11.22-18.59) | 54.58 (41.45-68.94) |  | 32.86(24.78-42.01) | 51.15(38.52-65.27) |
| East Asia | 243.32 (212.93-274.22) | 24.81 (21.77-27.83) |  | 656.34(559.06-774.82) | 31.10(26.57-36.49) |
| Eastern Europe | 206.34 (195.61-219.91) | 73.97 (70.00-78.97) |  | 262.04(231.31-295.07) | 83.08(73.38-93.58) |
| Eastern Sub-Saharan Africa | 57.05 (47.85-68.05) | 62.65 (52.53-74.68) |  | 131.62(110.22-153.53) | 64.64(54.63-74.64) |
| High-income Asia Pacific | 52.75 (51.33-53.87) | 25.95 (25.17-26.51) |  | 96.80(87.76-102.80) | 25.70(24.03-27.05) |
| High-income North America | 171.75 (166.53-176.34) | 52.72 (51.25-54.14) |  | 203.94(194.84-212.11) | 35.21(33.72-36.56) |
| North Africa and Middle East | 45.43 (38.25-52.71) | 23.17 (19.38-26.89) |  | 100.37(86.76-117.10) | 20.62(17.95-23.91) |
| Oceania | 1.79 (1.39-2.39) | 49.22 (38.00-65.22) |  | 4.53(3.42-6.32) | 52.56(40.46-71.89) |
| South Asia | 1178.63 (1043.57-1335.42) | 175.07 (152.58-198.85) |  | 2689.88(2316.87-3145.82) | 173.17(149.26-202.43) |
| Southeast Asia | 188.11 (169.41-204.01) | 65.46 (58.88-70.81) |  | 406.43(337.31-485.30) | 62.87(52.51-75.04) |
| Southern Latin America | 20.46 (19.63-21.24) | 43.82 (42.05-45.50) |  | 28.20(26.40-30.20) | 35.19(32.92-37.72) |
| Southern Sub-Saharan Africa | 23.23 (20.85-27.46) | 75.02 (66.60-89.59) |  | 40.66(37.02-45.17) | 65.17(59.56-72.29) |
| Tropical Latin America | 72.31 (69.90-74.68) | 71.20 (68.64-73.53) |  | 151.30(143.19-159.70) | 60.36(57.06-63.74) |
| Western Europe | 335.72 (327.35-343.84) | 65.97 (64.32-67.59) |  | 325.46(308.86-340.41) | 43.41(41.40-45.40) |
| Western Sub-Saharan Africa | 26.26 (22.49-30.51) | 26.34 (22.63-30.55) |  | 64.25(53.27-75.23) | 28.93(24.54-33.59) |
| DALYs (95% uncertainty interval), ASDR: age-standardized DALYs rate (95% uncertainty interval) | | | | | |

| Supplementary Table 3. The global incident cases and age-specific incidence rate of oral cancer by age in 1990 and 2019. | | | | | |
| --- | --- | --- | --- | --- | --- |
| Age groups | 1990 | |  | 2019 | |
|  | Incident cases (×10^3^) | Age-specific rate (1/10^5^) |  | Incident cases (×10^3^) | Age-specific rate (1/10^5^) |
| 0-14 | 0.81 (0.68-0.94) | 0.05 (0.04-0.05) |  | 1.09 (0.92-1.27) | 0.06 (0.05-0.06) |
| 15-19 | 1.18 (1.16-1.31) | 0.23 (0.20-0.25) |  | 1.62 (1.40-1.89) | 0.26 (0.23-0.31) |
| 20-24 | 1.71 (1.57-1.86) | 0.35 (0.32-0.38) |  | 2.87 (2.52-3.28) | 0.48 (0.42-0.55) |
| 25-29 | 2.39 (2.22-2.56) | 0.54 (0.50-0.58) |  | 4.46 (3.88-5.08) | 0.74 (0.64-0.84) |
| 30-34 | 3.76 (3.50-4.01) | 0.98 (0.91-1.04) |  | 7.71 (6.84-8.64) | 1.28 (1.14-1.44) |
| 35-39 | 6.54 (6.17-6.94) | 1.85 (1.75-1.97) |  | 12.78 (11.31-14.43) | 2.36 (2.09-2.67) |
| 40-44 | 9.70 (9.10-10.36) | 3.39 (3.18-3.62) |  | 17.90 (15.82-20.17) | 3.63 (3.21-4.09) |
| 45-49 | 14.04 (13.25-14.96) | 6.04 (5.70-6.44) |  | 27.00 (24.11-29.84) | 5.70 (5.09-6.30) |
| 50-54 | 20.01 (18.89-21.30) | 9.41 (8.88-10.02) |  | 39.10 (34.74-43.67) | 8.95 (7.95-10.00) |
| 55-59 | 23.67 (22.38-25.11) | 12.76 (12.07-13.54) |  | 48.33 (43.54-53.02) | 13.03 (11.74-14.29) |
| 60-64 | 25.63 (24.22-27.33) | 15.95 (15.07-17.01) |  | 50.06 (45.49-54.63) | 16.02 (14.55-17.48) |
| 65-69 | 22.82 (21.49-24.21) | 18.48 (17.40-19.60) |  | 49.43 (45.10-53.63) | 19.12 (17.44-20.74) |
| 70-74 | 16.89 (15.91-17.95) | 19.98 (18.83-21.24) |  | 40.85 (37.53-44.39) | 21.84 (20.06-23.73) |
| 75-79 | 14.03 (13.27-14.74) | 22.89 (21.64-24.04) |  | 32.03 (29.36-34.66) | 25.21 (23.11-27.28) |
| 80-84 | 7.67 (7.03-8.14) | 21.78 (19.96-23.10) |  | 20.58 (18.14-22.59) | 24.38 (21.49-26.76) |
| ≥85 | 4.78 (4.15-5.14) | 23.30 (20.25-25.09) |  | 17.29 (14.32-19.15) | 26.55 (21.99-29.41) |
| Incident cases (95% uncertainty interval), Age-specific incidence rate (95% uncertainty interval) | | | | | |

| Supplementary Table 4. The global deaths and age-specific mortality rate of oral cancer by age in 1990 and 2019. | | | | | |
| --- | --- | --- | --- | --- | --- |
| Age groups | 1990 | |  | 2019 | |
|  | Deaths (×103) | Age-specific rate (1/105) |  | Deaths (×103) | Age-specific rate (1/105) |
| 0-14 | 0.29 (0.24-0.34) | 0.02 (0.01-0.02) |  | 0.34 (0.28-0.40) | 0.02 (0.01-0.02) |
| 15-19 | 0.36 (0.32-0.41) | 0.07 (0.06-0.08) |  | 0.45 (0.38-0.54) | 0.07 (0.06-0.09) |
| 20-24 | 0.56 (0.51-0.62) | 0.11 (0.10-0.13) |  | 0.85 (0.74-0.99) | 0.14 (0.12-0.16) |
| 25-29 | 0.83 (0.77-0.90) | 0.19 (0.17-0.20) |  | 1.41 (1.22-1.64) | 0.23 (0.20-0.27) |
| 30-34 | 1.43 (1.32-1.55) | 0.37 (0.34-0.40) |  | 2.67 (2.34-3.03) | 0.44 (0.39-0.50) |
| 35-39 | 2.57 (2.38-2.76) | 0.73 (0.67-0.78) |  | 4.67 (4.11-5.28) | 0.86 (0.76-0.98) |
| 40-44 | 4.68 (4.32-5.07) | 1.63 (1.51-1.77) |  | 8.24 (7.22-9.36) | 1.67 (1.46-1.90) |
| 45-49 | 7.07 (6.54-7.68) | 3.04 (2.82-3.30) |  | 12.97 (11.49-14.65) | 2.74 (2.42-3.09) |
| 50-54 | 10.55 (9.81-11.40) | 4.96 (4.61-5.36) |  | 19.01 (16.82-21.45) | 4.35 (3.85-4.91) |
| 55-59 | 12.52 (11.69-13.48) | 6.75 (6.31-7.27) |  | 23.73 (21.42-26.32) | 6.40 (5.77-7.09) |
| 60-64 | 14.65 (13.57-15.87) | 9.12 (8.45-9.88) |  | 26.69 (24.12-29.46) | 8.54 (7.72-9.43) |
| 65-69 | 12.69 (11.79-13.68) | 10.27 (9.55-11.08) |  | 26.25 (23.92-28.63) | 10.15 (9.25-11.07) |
| 70-74 | 9.86 (9.12-10.64) | 11.66 (10.79-12.60) |  | 22.29 (20.37-24.44) | 11.92 (10.89-13.06) |
| 75-79 | 8.46 (7.89-9.00) | 13.79 (12.87-14.67) |  | 18.46 (16.86-20.11) | 14.53 (13.27-15.83) |
| 80-84 | 5.59 (5.12-5.99) | 15.87 (14.53-16.99) |  | 14.68 (12.97-16.20) | 17.39 (15.36-19.18) |
| ≥85 | 4.54 (3.93-4.93) | 22.14 (19.17-24.06) |  | 16.69 (13.69-18.43) | 25.63 (21.03-28.30) |
| Deaths (95% uncertainty interval), Age-specific mortality rate (95% uncertainty interval) | | | | | |

| Supplementary Table 5. The global DALYs and age-specific DALYs rate of oral cancer by age in 1990 and 2019. | | | | | |
| --- | --- | --- | --- | --- | --- |
| Age groups | 1990 | |  | 2019 | |
|  | DALYs (×10^3^) | Age-specific rate (1/10^5^) |  | DALYs (×10^3^) | Age-specific rate (1/10^5^) |
| 0-14 | 23.08 (18.93-27.14) | 1.32 (1.08-1.55) |  | 26.91 (22.48-31.51) | 1.37 (1.15-1.61) |
| 15-19 | 26.32 (23.20-29.61) | 5.06 (4.46-5.70) |  | 32.61 (27.67-39.10) | 5.26 (4.47-6.31) |
| 20-24 | 38.05 (34.76-42.00) | 7.72 (7.06-8.52) |  | 57.89 (50.61-67.07) | 9.65 (8.43-11.18) |
| 25-29 | 52.29 (48.15-56.79) | 11.81 (10.87-12.82) |  | 88.82 (76.97-103.11) | 14.67 (12.71-17.03) |
| 30-34 | 82.62 (76.36-89.84) | 21.42 (19.80-23.30) |  | 154.43 (135.48-175.18) | 25.66 (22.52-29.11) |
| 35-39 | 135.33 (125.58-145.50) | 38.36 (35.60-41.25) |  | 246.58 (217.16-278.86) | 45.58 (40.14-51.55) |
| 40-44 | 222.49 (205.65-241.26) | 77.72 (71.83-84.27) |  | 392.60 (344.59-446.09) | 79.56 (69.83-90.40) |
| 45-49 | 301.69 (279.26-327.79) | 129.81 (120.16-141.04) |  | 554.47 (491.82-625.80) | 117.03 (103.80-131.95) |
| 50-54 | 399.86 (371.63-431.86) | 188.09 (174.81-203.14) |  | 721.97 (638.17-812.59) | 165.28 (146.10-186.02) |
| 55-59 | 415.73 (388.26-446.89) | 224.22 (209.40-241.02) |  | 789.67 (712.93-874.21) | 212.84 (192.16-235.63) |
| 60-64 | 417.74 (387.40-452.55) | 260.04 (241.15-281.71) |  | 763.05 (690.37-841.14) | 244.15 (220.89-269.13) |
| 65-69 | 305.58 (283.70-329.64) | 247.46 (229.74-266.94) |  | 632.85 (576.40-689.54) | 244.74 (222.91-266.66) |
| 70-74 | 194.87 (180.48-210.43) | 230.58 (213.56-248.99) |  | 441.70 (404.39-483.69) | 236.09 (216.15-258.54) |
| 75-79 | 133.20 (124.34-141.83) | 217.26 (202.80-231.33) |  | 290.47 (265.31-316.29) | 228.62 (208.82-248.94) |
| 80-84 | 67.58 (61.88-72.42) | 191.89 (175.70-205.62) |  | 176.89 (156.99-194.61) | 209.53 (185.96-230.52) |
| ≥85 | 38.34 (33.31-41.67) | 187.00 (162.48-203.24) |  | 135.74 (112.91-149.84) | 208.48 (173.42-230.12) |
| DALYs (95% uncertainty interval), Age-specific DALYs rate (95% uncertainty interval) | | | | | |
